# A chemically modified solid-state sensor for magnesium(ii) ions and esomeprazole magnesium potentiometric assay†

**DOI:** 10.1039/d2ra06839g

**Published:** 2023-01-11

**Authors:** Ahmed S. Saad, Nahla S. Ismail, Noran S. Gaber, Eman S. Elzanfaly

**Affiliations:** a Analytical Chemistry Department, Cairo University, Faculty of Pharmacy Kasr El-Aini St 11562 Cairo Egypt ahmed.bayoumy@pharma.cu.edu.eg ahmedss_pharm@yahoo.com ahmed.s.saad@ejust.edu.eg +201004009443; b Medicinal Chemistry Department, PharmD Program, Egypt – Japan University of Science and Technology (E-JUST) New Borg El-Arab City 21934 Alexandria Egypt; c National Organization of Drug Control and Research Agouza Giza Egypt nahla.sayed@yahoo.com dr.noransaaid@hotmail.com; d Pharmaceutical Chemistry Department, Faculty of Pharmacy and Drug Technology, Egyptian Chinese University Cairo Egypt eman.elzanfaly@pharma.cu.edu.eg

## Abstract

The use of electrochemical sensors offers a simple, affordable solution with great reliability. Magnesium is a mineral that the body requires to function properly. It encourages preserving a stable pulse, strong bones, and healthy blood pressure. Herein, a novel ion-selective electrode using esomeprazole magnesium trihydrate as an ion-association complex was developed for magnesium(ii) ion determination in mineral water, drug substances, and pharmaceutical formulations. The electrode response was optimized in terms of plasticizer type, ion exchanger concentration, and membrane composition. To find the best sensor combination, the initial optimization research was performed using eight different sensors. A membrane containing 20% esomeprazole magnesium trihydrate, 36% carbon, and 44% *o*-Nitrophenyl Octyl Ether (NPOE) as a plasticizer yielded the best potentiometric response. The developed sensor demonstrated a Nernstian response with a slope of 29.93 ± 0.1 mV per decade in the concentration range of 1.41 × 10^−5^ mol L^−1^ to 1 × 10^−2^ mol L^−1^. Within a pH range of 5–8, it had a low detection limit of 4.13 × 10^−6^ mol L^−1^. When compared to the official method, there are no statistically significant differences.

## Abstract 2

The use of electrochemical sensors offers a simple, affordable solution with great reliability.

## Graphical abstract

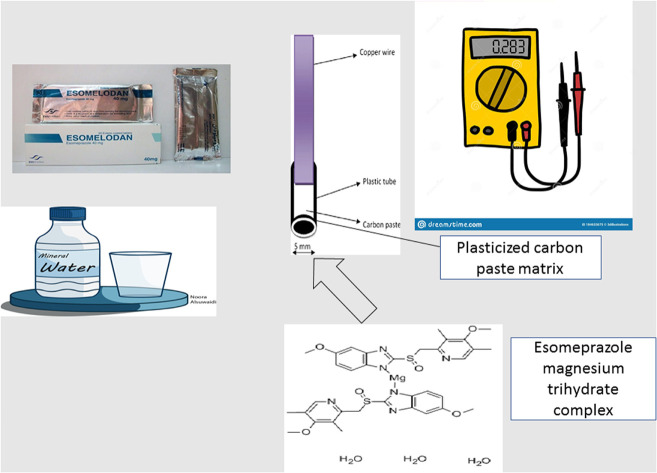

## 1. Introduction

Recently, the ion-selective electrode (ISE) has been regarded as one of the most outstanding contributions to analytical chemistry.^1,2^ It is a precise, fast, cost-effective, and sensitive technique for tracing ionizable analytes.^3^

ISEs have a wide range of applications, including food, feed, pharmaceutical industries,^4^ clinical assays,^5,6^ and environmental monitoring.^7,8^

Analysts continue to investigate novel electrode materials and new construction modules to meet the ever-increasing analytical demands.^9–12^

Magnesium is a trace element that is required for a variety of physiological processes. It is the second-most prevalent intracellular cation and a cofactor in over 300 enzymatic processes, including protein synthesis and energy consumption.^13–17^ A healthy immune system, a steady heartbeat, strong bones, and muscle function all depend on adequate magnesium levels.^18–20^ Disturbances in Mg balance are commonly noticed in hospitalized patients. A deficiency of magnesium can cause loss of appetite, fatigue, weakness, abnormal heartbeats, and muscle cramps, so it is very important to detect trace amounts of magnesium. ISEs with adequate selectivity for Mg(ii) are now available, which has recently encouraged several clinical trials.^21^ Literature reveals several methods for determination of magnesium ions as atomic absorption,^22^ colorimetry,^23^ and spectrophotometry.^24,25^ Little work has been done on the development of ISEs for magnesium. Only a few electrodes have been reported.^26–28^ However, most of them suffered from interference with alkaline earth metals, especially calcium ions, and a narrow working concentration range. The detection of magnesium(ii) ions suffers challenges. The potentiometric determination of the divalent magnesium(ii) ion requires a sensor with high selectivity, especially in the presence of high concentrations of calcium, sodium and potassium. In addition, the complex food supplement matrices may contain various compounds that may interfere with the Mg(ii) detection, such as lipids, proteins, carbohydrates, amino acids, vitamins, and other minerals.

**Fig. 1:**
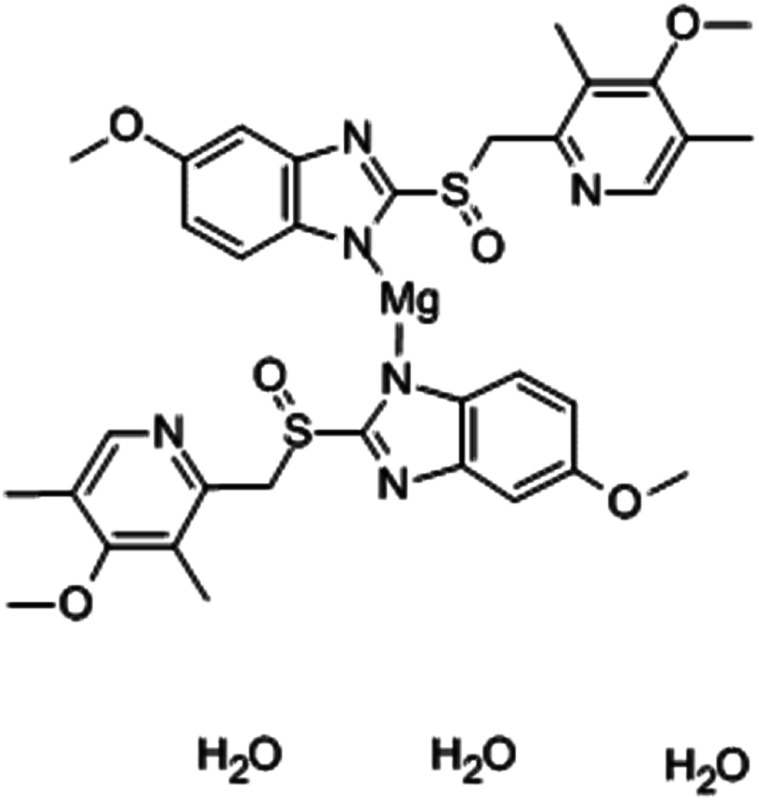
Chemical structure of esomeprazole magnesium trihydrate complex.

In our work, we introduce esomeprazole magnesium trihydrate (Fig. 1) as an ion-association complex for the development of a new sensor for the determination of magnesium(ii) ions in mineral water and pharmaceutical formulations. This complex is thought to be highly lipophilic, allowing it to be strongly retained in the hydrophobic membrane phase, ensuring the sensor's long lifetime. They also possess a polar moiety (magnesium(ii) ions) and a set of polar functional groups (hydroxyl groups) responsible for high selectivity.^29^

## 2. Experimental

### 2.1. Reagents and materials

All chemicals and reagents used were of the highest grade (ACS grade (≥95%)). Magnesium(ii) solutions were prepared using deionized water. All experiments used analytical grade reagents including pure graphite powders (Aldrich, 1–2 micron), dioctyl phthalate (DOP), tributyl phthalate (DBP), *o*-nitrophenyl octyl ether (*o*-NPOE), and hydrochloric acid. Sigma-Aldrich supplied ethylenediaminetetraacetic acid (EDTA). Sodium hydroxide was purchased from ADWIC. Merck (Germany) provided a standard magnesium(ii) solution. Esomeprazole magnesium trihydrate raw material and commercial Esomelodan tablets® 40 mg were kindly provided by EVA PHARM for pharmaceuticals and medical appliances.

### 2.2. Apparatus

We used a Jenway (3510) digital pH mV per meter for assessing potential measurements. All measurements were recorded at 25 ± 1 °C using an external reference saturated calomel electrode (SCE). The official atomic absorption spectrophotometric magnesium(ii) assay was conducted using a Solaar Atomic absorption spectrophotometer model (JE710572).

### 2.3. Standard solutions

Magnesium(ii) ion stock standard solution (1 × 10^−2^ mol L^−1^) was diluted using deionized water to prepare working standard solutions with concentration ranging from 1 × 10^−6^ mol L^−1^ to 1 × 10^−3^ mol L^−1^.

### 2.4. Preparation of modified carbon paste electrode

**Fig. 2:**
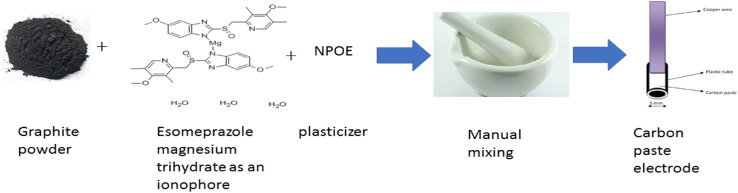
Diagram for the fabrication process of esomeprazole magnesium sensor.

The detecting carbon paste electrode was produced by manually combining esomeprazole magnesium trihydrate, graphite, and plasticizer in precisely measured amounts in a ratio of (0.5 : 1 : 1.2) respectively to create a uniform paste. The prepared paste was inserted into the opening in the sensor body. The electrode surface was buffed with filter paper to create a reproducible, shiny working surface (Fig. 2). Then, without the need for preconditioning, potentiometric measurements were made directly on this surface.

### 2.5. Sensor calibration

The stock standard magnesium solution 1 × 10^−2^ mol L^−1^ was prepared using deionized water. From the stock solution, successive dilutions in the range of (1 × 10^−6^ mol L^−1^ to 1 × 10^−3^ mol L^−1^) were prepared. The esomeprazole magnesium sensor and the reference calomel electrode were submerged in the solutions at 25 ± 1 °C. Calibration curves were established by plotting the measured potential values against the negative logarithmic values of magnesium concentrations (pMg).

### 2.6. Selectivity measurement

The potentiometric selectivity coefficients (*K*^pot^_Mg,*j*_) for various interfering ions were estimated using the separate solution method (SSM)^30^ and the matched potential method (MPM).^31,32^ In the SSM, the potential difference (*E*) between the working electrode and the reference electrode was recorded in 1 × 10^−3^ mol L^−1^ Mg standard solution (*E*_Mg_) and 1 × 10^−3^ mol L^−1^ solution of the interfering ion (*E*_*j*_). The selectivity coefficients (*K*^pot^_Mg,*j*_) were calculated using the Nickolsky–Eisenman equation:1
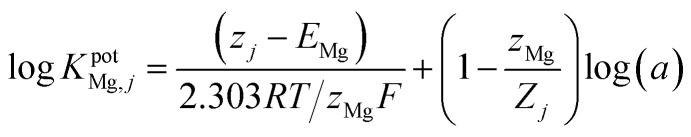


The potentiometric selectivity coefficient in the matched potential method is defined as the activity ratio of primary and interfering ions that produce the same potential change under the same circumstances. At first, a known activity of standard Mg solution 
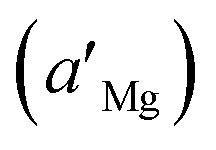
 is added to a fixed activity reference solution (*a*_Mg_) and induces a change in the potential. We determined the activity of the interfering ion (*a*_*j*_) that induced the same change in the potential when added to the reference solution of the same Mg activity (*a*_Mg_). The selectivity coefficient was calculated using the following equation:2
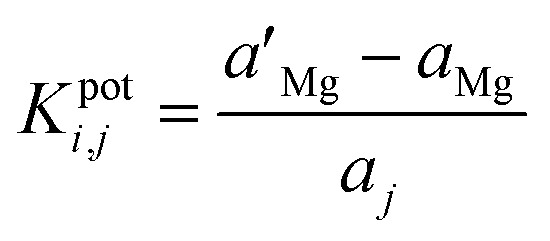


### 2.7. Effect of pH

The pH effect on sensor response was investigated for magnesium(ii) solutions (1.0 × 10^−3^ and 1.0 × 10^−4^ mol L^−1^). Small increments of HCl and NaOH solutions (0.1–1 mol L^−1^) were used to adjust the pH. The potential (mV) variation over the pH range (1–10) was recorded in order to plot the potential-pH profile for the optimised sensor.

### 2.8. Response time and lifespan

The dynamic response time was measured while the electrode was immersed in a series of magneium(ii) ion standard solutions ranging from 1.0 × 10^−4^ mol L^−1^ to 1.0 × 10^−2^ mol L^−1^. The response time was calculated by measuring the time required to reach the equilibrium potential of ±1 mV. The paste was kept in a sealed plastic falcon tube and kept in the refrigerator at 4–5 °C when not in use. The stored paste was taken out of the fridge before measurements and allowed to reach room temperature before being packed into the Teflon electrode body. Regular calibrations were carried out (every 15 days). To determine the sensor lifespan, we plotted the slopes against time.

### 2.9. Analytical application

#### 2.9.1. Drug substance

Stock magnesium solution (1 × 10^−3^ mol L^−1^) was prepared by dissolving an accurately weighed 76.7 mg esomeprazole magnesium trihydrate in 5 ml 1 N HCl and completing the volume to 100 ml with deionized water. Two volumes (5 ml and 10 ml) of stock solution were added to 50 ml of deionized water and the pH was adjusted to 6 using small volumes of NaOH (0.1–1 mol L^−1^). The solutions were titrated by 1 × 10^−3^ mol L^−1^ EDTA. The *S*-shaped curves with a first plots were used to determine the endpoints.

#### 2.9.2. Drug dosage form

Twenty tablets of (Esomelodan 40 mg) B.N. (602483) were powdered. An amount equivalent to the average of one tablet was digested with 5 ml of 1 N HCl and completed to 25 ml using deionized water. A volume of 21.5 ml from the previous solution was diluted to 50 ml using deionized water to obtain a stock solution concentration of 1 × 10^−3^ mol L^−1^. Two volumes (5 ml and 10 ml) of the prepared solution were added to 50 ml of deionized water. The pH of solutions was adjusted to 6 titrated by 1 × 10^−3^ mol L^−1^ EDTA. The first derivative plots were used to determine the endpoints. The results obtained by the proposed ISE were compared with those obtained by flame AAS.^33^

#### 2.9.3. Analysis of mineral water

A volume of 1000 ml of Nestle mineral water (claimed to contain 6.72 mg L^−1^ magnesium) was adjusted to pH 6, titrated using 1 × 10^−1^ M EDTA, and the end point was determined potentiometrically. The obtained results were compared to those obtained by the official atomic absorption method.^34^

## 3. Results and discussion

In addition to the advantages of portability and simplicity, ion-selective electrodes (ISE) have shown apparent benefits for analytical laboratories, including low analysis costs, minimal sample preparation, and quick analysis times. Electrochemists are constantly looking for new electrode materials and sensor assemblies to create robust sensors with improved performance traits.

In graphite, each carbon atom bonds covalently to three adjacent carbon atoms to form a layer of hexagonal arrays. The unbound carbon electrons (an electron from each carbon atom) form an electron sea. The electron sea loosely bounds the layers together by van der Waals interactions.^35^ Graphite intercalates the esomeprazole magnesium association complex between its layers. Charge transfer occurs between the complex and graphite to yield electrically conductive material in graphite intercalation compounds.^36^ The plasticized carbon paste contains esomeprazole magnesium complex, the difference in magnesium activity between the carbon paste and the aqueous phase generates a force that drives magnesium to partition into the aqueous phase. Interfacial charge separation occurs as the positive magnesium ions cross the interface. In the absence of current, a phase boundary potential (*E*_PBP_) builds up to counterbalances this driving force^37^ (Fig. S1†).3
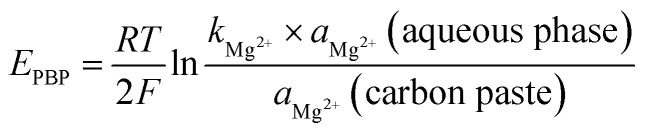


in which *a*_Mg^2+^_ is the magnesium ion activity, *R* is the gas constant, *T* is the absolute temperature, *F* is the Faraday constant, and *k*_Mg^2+^_ is a constant that includes the standard Gibb's free energy of magnesium ion transfer. The esomeprazole acts also as an ionophore that selectively bounds magnesium to minimize the Gibb's free energy of magnesium ion transfer and minimize the interference from other sample solution ions and keep constant magnesium activity within the carbon paste.^37^ Thus, the magnesium ion activity in the aqueous phase solely affects the phase boundary potential as follows:4
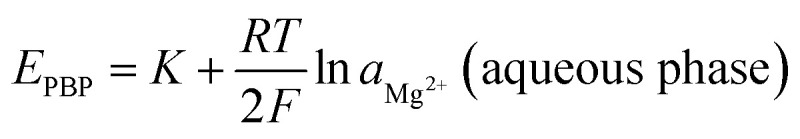
where *K* is the electrode constant.

The esomeprazole magnesium limited solubility and strong cation exchanging capacity protects against leaching and Mg(ii) efflux into the aqueous sample solution. This maintains a constant Mg(ii) concentration within the sensor matrix over long periods of time and across a wide pH range.^38^

The current work investigated the ability to use esomeprazole magnesium trihydrate complex in the fabrication of Mg electrode for several measurements in mineral water and pharmaceutical formulations. The synthesized electrode showed a Nernstian slope (29.93 ± 0.01 mV per decade) with a fast response time (8–10 s) over a wide linear range (1.41 × 10^−5^ mol L^−1^ to 1 × 10^−2^ mol L^−1^). The electrode can function effectively in the pH range of 5–8 with a detection limit of (4.13 × 10^−6^ mol L^−1^).

Sensor selectivity, sensitivity, linear range, detection limit, and stability of an ISE are influenced by factors such as the graphite-to-plasticizer ratio,^39^ the nature of the plasticizer,^39,40^ and the amount of esomeprazole–Mg complex in the paste. We studied the effect of each of these factors on the sensor performance to reach the optimum sensor composition.

### 3.1. Optimization of the paste composition

The optimization study's goal is to determine the best sensor recipe with a Nernstian slope, a large linear range with adequate sensitivity and selectivity, a quick stable response, and a long lifetime. These performance parameters are directly influenced by the sensor composition.

**Fig. 3:**
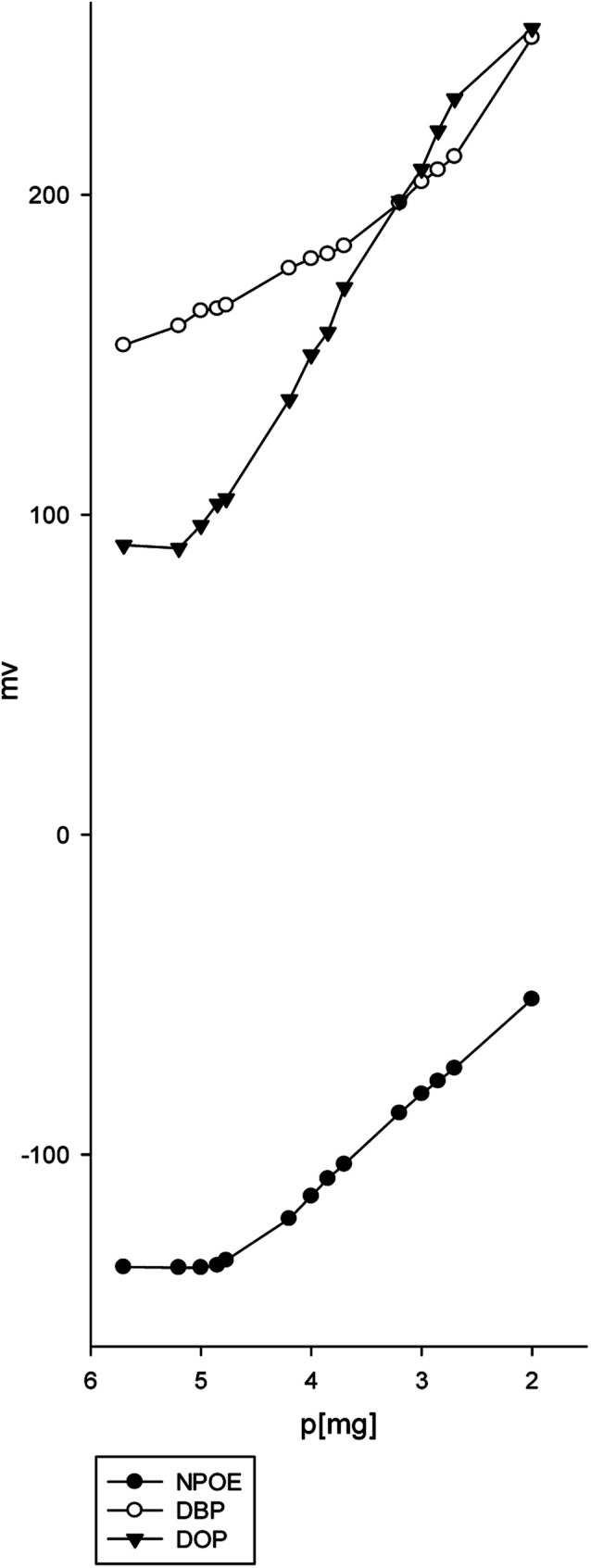
Electrode response for determination of magnesium(ii) solution using different plasticizers *o*-NPOE, DBP, and DOP.

The nature of the plasticizer can influence the dielectric constant of the membrane, ionophore mobility, and the state of the ligand.^41^ Three sensors with various plasticizers (DBP, DOP, and NOPE) were fabricated to evaluate the ion-selective characteristics. It is clear that the *o*-NPOE plasticizer is more effective than DPB and DOP owing to its higher dielectric constant.^41–43^ In addition, *o*-NPOE dissolves the complex that improves both permittivity and ion exchanger site mobility to give the highest sensitivity and selectivity (Fig. 3).

**Fig. 4:**
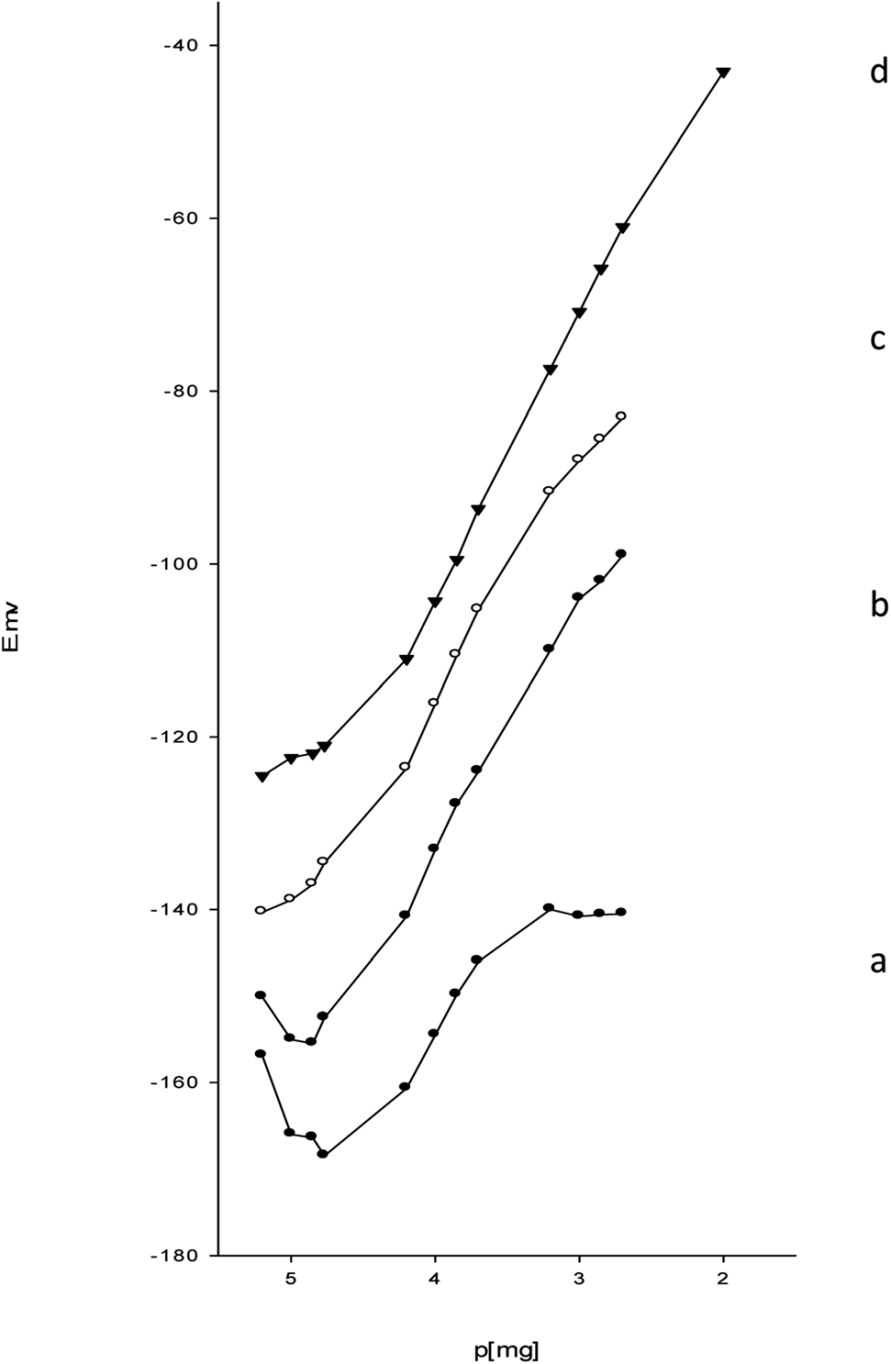
Calibration graph for determination of magnesium(ii) metal using (a) 5%, (b) 10%, (c) 15%, and (d) 20% esomeprazole magnesium trihydrate, 36% C, and 44% NPOE sensor.

We studied various graphite-to-plasticizer ratios. The optimum response resulted when the sensor matrix comprised graphite and NPOE in the ratio of 1.0 : 1.2 (Table 1). The effect of different concentrations of the esomeprazole magnesium trihydrate complex on the response of the electrode was studied in (Fig. 4). The 20% ion pair showed the lowest detection and quantification limits and the best near-Nernstian slope. A comparison of all the sensors revealed that the paste containing 20% esomeprazole–Mg complex, 36% graphite, and 44% *o*-NPOE exhibited the best response characteristics with a low detection limit.

**Table 1:** Composition, slope, linear range, and detection limits of calibration curves of magnesium sensors

Paste no.	Composition% (w/w)					Response parameters^a^		
	Ion pair	Graphite	Plasticizer			Slope ± RSD mV per decade	Linear range (mol L^−1^)	LOD (mol L^−1^)
			*o*-NOPE	DOP	DBP			
1	10%	41%	50%	—	—	27.07 ± 0.013	1.69 × 10^−5^ to 1.99 × 10^−3^	9.10 × 10^−6^
2	15%	38%	46%	—	—	26.54 ± 0.009	1.69 × 10^−5^ to 1.99 × 10^−3^	4.46 × 10^−6^
3	5%	43%	52%	—	—	19.30 ± 0.014	1.69 × 10^−5^ to 6.30 × 10^−4^	8.70 × 10^−6^
4	20%	36%	—	44%	—	56.01 ± 0.0008	1.69 × 10^−5^ to 1.00 × 10^−2^	1.99 × 10^−6^
5	20%	36%	—	—	44%	24.44 ± 0.0124	1.69 × 10^−5^ to 1.99 × 10^−2^	3.10 × 10^−5^
6	20%	40%	40%	—	—	30.10 ± 0.006	1.69 × 10^−5^ to 1.00 × 10^−2^	5.01 × 10^−6^
**7** b	**20%**	**36%**	**44%**	**—**	**—**	**29.93±0.01**	**1.41 × 10^−5^ to 1.00 × 10^−2^**	**4.13 × 10^−6^**
8	20%	39%	41%	—	—	27.28 ± 0.006	1.69 × 10^−5^ to 1.00 × 10^−2^	6.30 × 10^−6^

a Average of three determinations.

b The selected sensor.

The IUPAC recommendations were followed to evaluate the characteristic performance parameters of the optimized sensor (Table S1†).

### 3.2. Effect of pH on the electrode potential

The influence of the pH of magnesium solutions on the potential response values of the esomeprazole magnesium electrode was studied at the concentration of 1.0 × 10^−3^ and 1.0 × 10^−4^ mol L^−1^ at different pH (1.0–10.5). The pH of the solutions was adjusted by the addition of diluted HCl and/or NaOH solutions (0.1–1.0 mol L^−1^). The potential change at each pH value was reported. Plot the mV readings against the pH values for the different concentration (Fig. S2†). It is obvious that the pH range of 5–8 will yield the optimal performance for the constructed electrode. The change of potential at higher pH values may be due to the precipitation of magnesium hydroxide, while at lower pH values, H^+^ ions contribute to the charge transport process through the membrane, causing pronounced interference.^44^

### 3.3. Dynamic response and lifetime

The dynamic response time^44^ for the electrode was tested by recording the time needed to achieve a steady state (within ±1 mV) after successive immersions of the electrode in 1.0 × 10^−4^, 1.0 × 10^−3^, and 1.0 × 10^−2^ mol L^−1^ magnesium(ii) solutions. The static response time for the fabricated electrode thus obtained was within 8 to 10 seconds. The potential time plot for the response of the esomeprazole magnesium electrode is shown in Fig. S3.† The life span of the prepared paste was tested every 5 days over 5 months. The significant changes in the slope of the electrode started after 4 months, and then a slight gradual decrease in the slope from (30.2 to 26.3 mV per decade) was observed, as shown in Fig. S4.† The sensor efficiency drops off after 100 days as a result of decomposition and leaching of the ionophore from the membrane.^45^ The lifetime of the electrode surface was 6 experiments. After that, a new surface for electrode measurements can be achieved by removing a very small amount of the surface and polishing it with filter paper till a smooth surface is obtained.

### 3.4. Selectivity of the sensor

The selectivity coefficients illustrate how effectively the fabricated sensor discriminates between the primary ion and other ions present in the solution (Table S2†). The outcomes expressed adequate selectivity and sensitivity to magnesium ions. The selectivity coefficient was calculated using a separate solution method (SSM) for charged species and a matched potential method (MPM) for uncharged species.

### 3.5. Analytical application

**Fig. 5:**
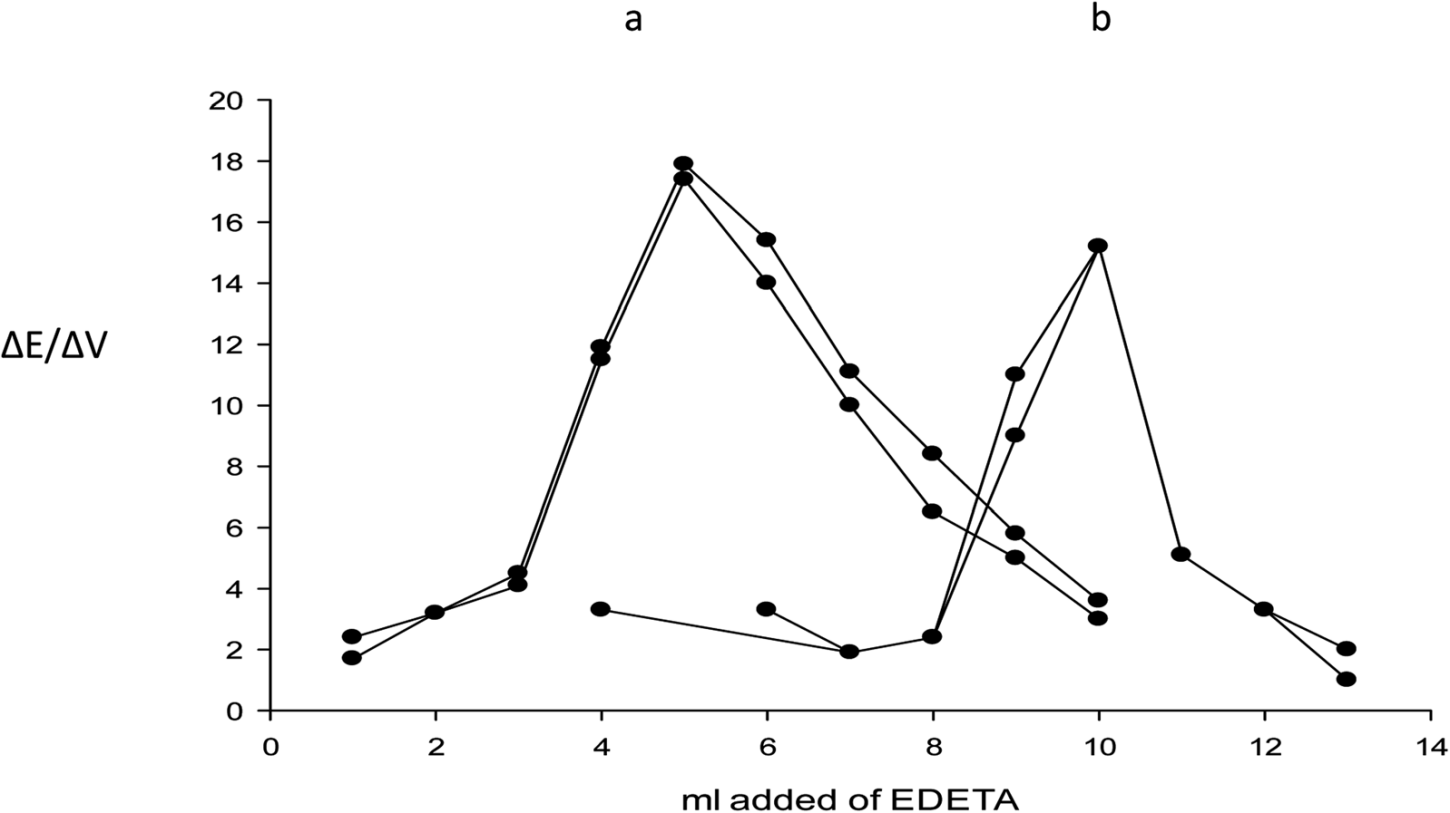
Potentiometric titration of (a) 3.8 mg of esomeprazole magnesium drug substance and drug product, and (b) 7.6 mg of esomeprazole magnesium drug substance and drug product against 10^−3^ M EDTA using the optimized esomeprazole magnesium sensor.

The fabricated electrode was applied successfully for the direct assay of magnesium(ii) in the drug substance and finished products, also it is used indirectly for the assay of esomeprazole magnesium trihydrate by potentiometric titration using EDTA as a titrant. The end point was determined using the first derivative method. The potential data was plotted against the volume of EDTA as shown in Fig. 5. The results of the recovery of magnesium(ii) ions and esomeprazole magnesium trihydrate using the potentiometric titration method were evaluated statistically and compared with data obtained from the pharmacopeial method using flame AAS and HPLC.^33^ The accuracy and precision were tested using three determinations of concentration, 10^−3^ M for both the drug substance and the dosage form. The mean recovery obtained was within ±2.0% for the drug substance. The relative standard deviation was ≤2.0% which indicates reasonable repeatability and reproducibility of the proposed method. The precision and accuracy of the assay of magnesium(ii) in drug substances, esomelodan tablets, and mineral water using the fabricated electrode were summarized in Table 2. The *T*-test and *F*-test^46,47^ indicate there is no significant difference between the official and proposed methods.

**Table 2:** Accuracy and precision for quantification of magnesium(ii) in the drug substance and dosage form (esomelodan) tablets using esomeprazole magnesium sensor^a^

Sample	Analyte	Recovery% ± RSD%		*F*-test (19.16)	*T*-paired test (4.30)
		Potentiometric titration	Official method		
Drug substance	Mg^2+^ content	99.17 ± 1.04	101.05 ± 0.60	1.07	1.26
	Esomeprazole Mg content		100.28 ± 0.30 (ref. 48)		
Esomelodan tablets (40 mg)	Mg^2+^ content	101.65 ± 0.39	92.74 ± 0.10	1.18	2.49
	Esomeprazole Mg content		99.76 ± 1.50 (ref. 49)		
Nestle® mineral water	Mg^2+^ content	101.19 ± 0.28	101.7 ± 0.42	1.29	0.77

a Significance level 5%, number of sample = 3.

## 4. Comparison of the magnesium selective sensors

The comparison of the performance characteristics of the fabricated electrode with other reported ones is presented in Table 3. As can be seen, the linear range and lifetime are superior to other reported magnesium ion selective electrodes.

**Table 3:** Comparison of response characteristics of the esomeprazole-Mg sensor* with some previously reported ones

S. no	Response range (mol L^−1^)	Slope (mV per decade)	Detection limit (mol L^−1^)	Response time (s)	Life time (weeks)	Type of ionophore	Reference
1	1.0 × 10^−5^ to 1.0 × 10^−1^	31	—	15	16	Benzo-15-crown-5(i)	27
2	9.4 × 10^−6^ to 1.0 × 10^−1^	29.2	—	13	20	Magnesium–tetrazaporphyrin	28
3	3.2 × 10^−5^ to 1.0 × 10^−1^	30	—	—	4	1,2-Bis(diarylphosphine oxide) benzene	50
4	6.0 × 10^−4^ to 1.8 × 10^−3^	28.6	0.1 × 10^−5^	3	1	ETHT 5504	51
5	1.0 × 10^−5^ to 5.0 × 10^−2^	28.2	6.28 × 10^−6^	10	8	Polypyrrole doped with titan yellow dye	52
6	1.0 × 10^−5^ to 1.0 × 10^−1^	29.2	1.0 × 10^−5^	10	4	4,5-Bis(benzoylthio)-1,3-dithiole-2-thione (Bz_2_dmit)	53
7	1.0 × 10^−5^ to 1.0 × 10^−1^	29.5	1.4 × 10^−5^	10	—	Schiff base chitosan and 5-nitro isatin	54
8*	1.41 × 10^−5^ to 1 × 10^−2^	29.9	4.13 × 10^−6^	8–10	16	Esomeprazole–magnesium	Proposed electrode

## 5. Conclusion

The study presenting esomeprazole magnesium trihydrate as a new ion-association complex to be incorporated in the manufacture of ion selective electrode for magnesium(ii) assay. The fabricated electrode is used for the determination of magnesium(ii) ion in mineral water and pharmaceutical formulations.

The good recoveries and low standard deviations obtained indicated the high accuracy and precision of the proposed method. The working concentration range of the sensor is from 1.41 × 10^−5^ to 1 × 10^−2^ mol L^−1^. The membrane was prepared using a ratio of (20% esomeprazole–Mg complex, 44% *o*-NOPE, and 36% graphite). The detection limit was found to be 4.13 × 10^−6^ with a slope of 29.93 ± 0.01 mV per decade. The electrode possesses stable potential at a pH range of 5–8 with a response time of 8–10 s. The selectivity of the electrode is quite good towards magnesium(ii) over other cations with a lifetime of 16 weeks. In addition, the sensor can be used as an indicator electrode for both the direct determination of magnesium(ii) and indirect determination of esomeprazole-mg trihydrate in both drug substances and drug products using EDTA as a titrant.

## Conflicts of interest

The authors declare no conflict of interest.

## Supplementary Material

RA-013-D2RA06839G-s001
