# Supplementary material for: A chemically modified solid-state sensor for magnesium(ii) ions and esomeprazole magnesium potentiometric assay
Source: RSC Adv. 2023 Jan 11;13(3):1995–2003. doi: 10.1039/d2ra06839g (PMC9832439; doi:10.1039/d2ra06839g)
Supplement: RA-013-D2RA06839G-s001 [file RA-013-D2RA06839G-s001.pdf]

Electronic Supplementary Material (ESI) for RSC Advances.
This journal is © The Royal Society of Chemistry 2023

Electronic Supplementary Material (ESI)

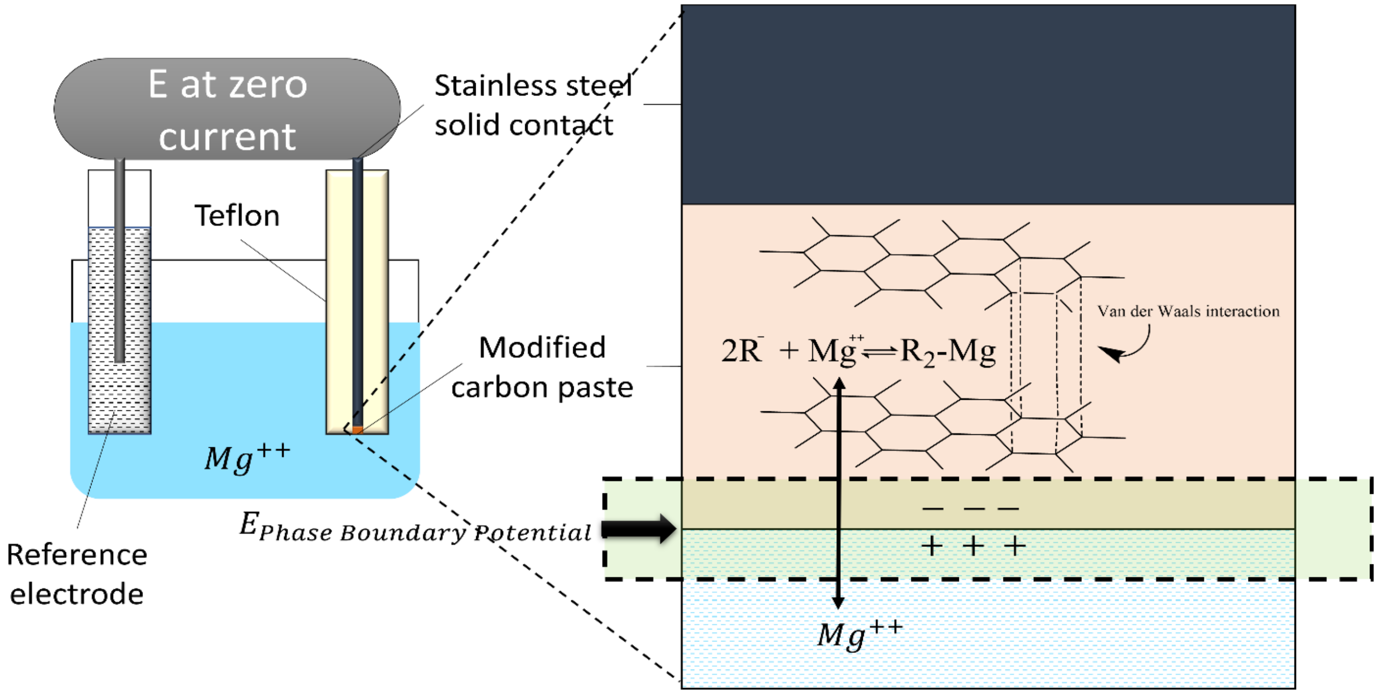

Fig. S1 Diagram for magnesium(II) ions detection using esomeprazole magnesium carbon paste electrode

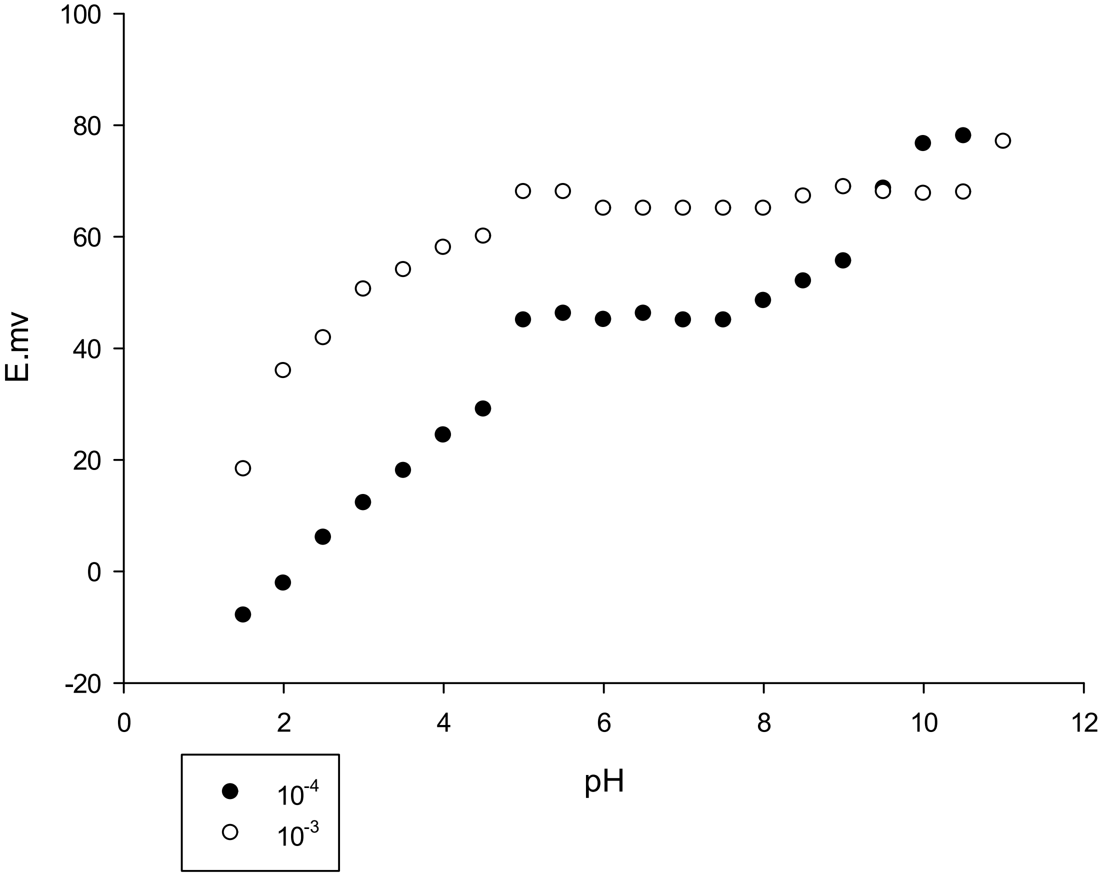

Fig. S2 Effect of pH on 1×10^{-3} and 1×10^{-4} mol L^{-1} Magnesium(II) solutions on the potential response of the esomeprazole-mg sensor

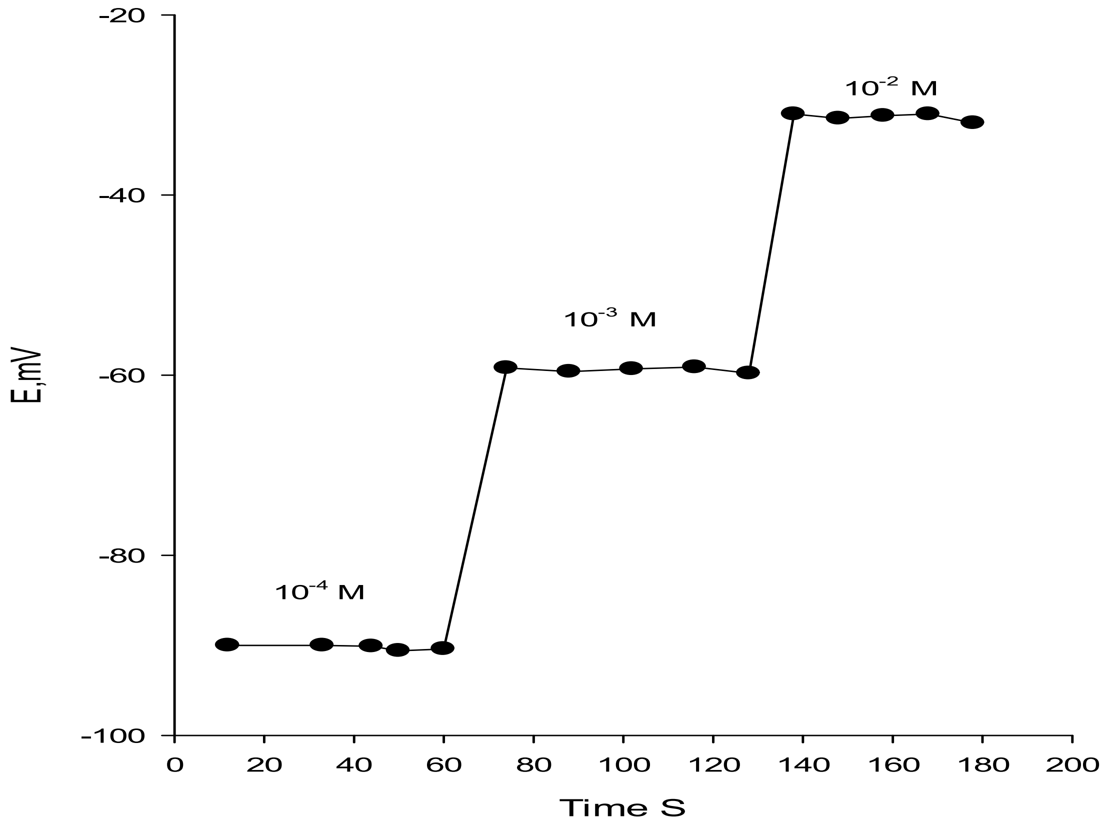

Fig. S3 Potential-Time response of the esomeprazole magnesium sensor using Magnesium(II) solutions 1×10^{-4} to 1×10^{-2} mol L^{-1}

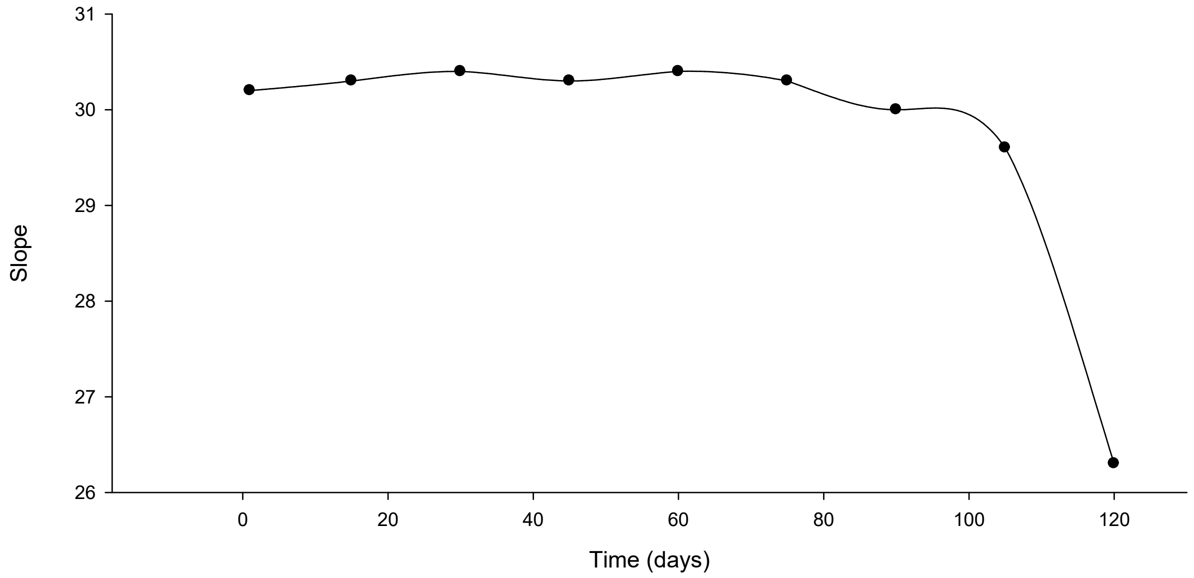

Fig. S4 Life span of the esomeprazole magnesium sensor

Table S1 Response characteristics of the optimized sensor[(a)].

Parameters	Optimized sensor response[(a)]
Slope (mV $decade^{-1}$)	29.93 ± 0.10
Correlation coefficient (r)	0.996
LOD (mol L^{-1})	4.13×10^{-6}
Linear range (mol L^{-1})	$1.41\times10^{-5} - 1.00\times10^{-2}$
Accuracy (recovery % ± %RSD)	100.5±0.39
Working pH range	5.0–8.0
Response time (s)	8-10
Life span (months)	4

[a)] The sensor consists of 20% esomeprazole mg, 36% graphite, and 44% NOPE.

Table S2 Selectivity coefficient values for the esomeprazole-mg sensor

Interfering ions	$\log K_{Mg,j}^{pot}$
Fe^{3+}	-4.89
Zn^{2+}	-1.81
Cr^{36}	-3.06
Se^{6+}	-1.17
Ni^{3+}	-1.26
Ca^{2+}	-2.06
K^{+}	-1.18
Na^{+}	-1.18
Mo^{2+}	-2.73
Cu^{2+}	-2.96
Co^{3+}	-3.15
Na-CMC	-2.47
Lactose	-2.42
Starch	-2.39
MC.Cellulose	-2.58
Talc	-2.18
Gelatin	-1.9
Sucrose	-3.49
L-Glutamine	-2.17
B-Alanine	-1.54
L-Threonine	-2.69
